# Intravenous paracetamol in comparison with ibuprofen for the treatment of patent ductus arteriosus in preterm infants: a randomized controlled trial

**DOI:** 10.1007/s00431-020-03780-8

**Published:** 2020-09-04

**Authors:** Carlo Dani, Gianluca Lista, Silvia Bianchi, Fabio Mosca, Federico Schena, Luca Ramenghi, Enrico Zecca, Giovanni Vento, Chiara Poggi, Valentina Leonardi, Diego Minghetti, Maria Teresa Rosignoli, Fabrizio Calisti, Alessandro Comandini, Agnese Cattaneo, Paola Lipone

**Affiliations:** 1 Department of Neuroscience, Psychology, Drug Research and Child Health, Careggi University Hospital of Florence, Largo Brambilla 3, 50134 Florence, Italy; 2 Division of Neonatology, Careggi University Hospital of Florence, Largo Brambilla 3, 50134 Florence, Italy; 3 Division of Neonatology, “V. Buzzi” Children Hospital of Milan, Via Castelvetro 22, 20154 Milan, Italy; 4 Fondazione IRCCS Ca’ Granda Ospedale Maggiore Policlinico Milan – NICU, Via Della Commenda 12, 20122 Milan, Italy; 5 Department of Clinical Sciences and Community Health, University of Milan, Via Della Commenda 12, 20122 Milan, Italy; 6 Department of Neonatology Obstetrics and Neuroscience, G. Gaslini Children’s University Hospital of Genova, Via Gerolamo Gaslini 5, 16147 Genoa, Italy; 7 Division of Neonatology, Catholic University of Rome, Largo Agostino Gemelli 8, 00168 Rome, Italy; 8 Angelini Pharma S.p.A, Viale Amelia 70, 00181 Rome, Italy

**Keywords:** Paracetamol, Patent ductus arteriosus, Preterm infant

## Abstract

Our aim was to assess the efficacy and safety of intravenous (i.v.) paracetamol vs. i.v. ibuprofen for the treatment of hemodynamically significant patent ductus arteriosus (hsPDA) in preterm infants. This is a multicenter randomized controlled study. Infants with a gestational age of 25^+0^–31^+6^ weeks were randomized to receive i.v. paracetamol (15 mg/kg/6 h for 3 days) or i.v. ibuprofen (10-5-5 mg/kg/day). The primary outcome was the closure rate of hsPDA after the first treatment course with paracetamol or ibuprofen. Secondary outcomes included the constriction rate of hsPDA, the re-opening rate, and the need for surgical closure. Fifty-two and 49 infants received paracetamol or ibuprofen, respectively. Paracetamol was less effective in closing hsPDA than ibuprofen (52 vs. 78%; *P* = 0.026), but the constriction rate of the ductus was similar (81 vs. 90%; *P* = 0.202), as confirmed by logistic regression analysis. The re-opening rate, the need for surgical closure, and the occurrence of adverse effects were also similar.

*Conclusions*: Intravenous paracetamol was less effective in closing hsPDA than ibuprofen, but due to a similar constriction effect, its use was associated with the same hsPDA outcome. These results can support the use of i.v. paracetamol as a first-choice drug for the treatment of hsPDA.

*Trial registration***:** Clinicaltrials.gov: NCT02422966, Date of registration: 04/09/2015; EudraCT no: 2013-003883-30.

**Table:** 

**What is Known:** *• The successful closure of patent ductus arteriosus with oral paracetamol has been recently reported in several preterm infants, but only one randomized controlled study investigated the efficacy of intravenous paracetamol.*	
**What is New:** *• Intravenous paracetamol is less effective in closing hsPDA than ibuprofen, but have a similar constriction effect.* *• These results can support the use of i.v. paracetamol as a first-choice drug for the treatment of hsPDA.*

**Electronic supplementary material:**

The online version of this article (10.1007/s00431-020-03780-8) contains supplementary material, which is available to authorized users.

## Introduction

Patent ductus arteriosus (PDA) is a frequent complication in preterm infants with respiratory distress syndrome (RDS), and 60–70% of preterm infants of < 28-week gestation receive medical and/or surgical treatment for PDA [1]. The proper management of PDA is the subject of lively debate because randomized controlled trials (RCTs) of PDA closure using non-steroidal anti-inflammatory drugs (NSAIDs) often failed to demonstrate relevant benefits in preterm infants [2]. However, a persistent left-to-right shunt through the ductus arteriosus (DA) complicating RDS has been associated with a worsening of respiratory failure, lowering of survival rate, increased risk of intraventricular hemorrhage (IVH), and bronchopulmonary dysplasia (BPD) [1, 3–6]. Therefore, the closure of PDA is indicated before a significant left-to-right shunting occurs.

The current treatment of PDA encompasses two steps: the first is the pharmacological treatment with a NSAID; the second, in case of medical treatment failure, is the surgical ligation, which should be avoided, if possible, due to the associated severe complications [7]. Standard medical therapy for the PDA closure mainly involves either indomethacin or ibuprofen. Both are successful in promoting the ductal closure in 70–80% of cases [8, 9]. However, these drugs can cause severe adverse effects including gastrointestinal perforations, acute renal failure, and bleeding disorders [8, 9]. Therefore, although ibuprofen appears to be at present the drug of choice for PDA pharmacological closure, due to its fewer side effects compared with indomethacin [9], it does not represent the ideal drug because of its sub-optimal safety profile [9] and because of its approximately 30% failure rate [10, 11].

Successful closure of PDA with oral paracetamol has been reported by RCTs in several preterm infants [12–14]. Moreover, the safety profile of paracetamol has been found to be better than that of indomethacin and ibuprofen with a lower rate of gastrointestinal and renal adverse effects [15, 16] and no detrimental effect on cerebral oxygenation [17]. However, the effectiveness of i.v. paracetamol has recently been questioned, since only one RCT investigated the efficacy of i.v. paracetamol in closing PDA [14] and retrospective studies found a lower rate of PDA closure [18] or constriction (i.e., lower rate of closed or not hsPDA) in comparison with indomethacin [18] and ibuprofen [18, 19], especially in the most immature infants (gestational age < 26 weeks).

On this basis, we deemed a further study necessary to confirm or confute previous findings on the efficacy of i.v. paracetamol and to learn more about its possible side effects. Thus, the present study assessed the efficacy and safety of i.v. paracetamol in comparison with i.v. ibuprofen for the treatment of hsPDA in preterm infants.

## Materials and methods

This is a multicenter randomized controlled study (Clinicaltrials.gov: NCT02422966; EudraCT no: 2013-003883-30) involving five Neonatal Intensive Care Units in Italy. The study was approved by institutional review board and relevant authorities according to local regulations.

### Study population

Inclusion criteria were gestational age of 25^+0^–31^+6^ weeks, obtained parental consent, and echocardiographic evidence of hsPDA between 24 and 72 h of life. The diagnosis of hsPDA was made by echocardiographic demonstration of a ductal left-to-right shunt, with a left atrium-to-aortic root ratio > 1.3 or a ductal size > 1.5 mm and excluding the cases in which the closing flow pattern suggested a restrictive PDA [20, 21]. Exclusion criteria were major congenital malformations, fetal hydrops, life-threatening infection defined as positive blood culture sampled at birth, echocardiographic evidence of pulmonary hypertension, and grade ≥ 3 IVH; serum creatinine concentration > 1.5 mg/dL, urine output < 1 mL/kg/h during a 24-h collection period or urine output < 0.5 mL/kg/h during the first 24 h of life; platelet count < 50,000/mm^3^; major bleeding, as revealed by hematuria, or blood in the tracheal aspirate, gastric aspirate, or stools or consistent blood oozing from puncture sites; and severe liver failure, defined as elevated liver enzymes (ALT, AST) > 2 times the upper boundary of the normal range (ALT 6–50 U/L; AST 35–140 U/L) [22].

### Study design

Infants were randomly assigned in blocks to a treatment group in 1:1 ratio. Patients in group I received 15 mg/kg/6 h of i.v. paracetamol (Tachipirina®, Angelini S.p.A., Ancona, Italy) for 3 days [12–14]. Patients in group II received an initial dose of 10 mg/kg, followed by 5 mg/kg after 24 and 48 h of i.v. ibuprofen (Pedea®, Orphan Europe S.A.R.L., Puteaux, France). Both drugs were infused continuously over a period of 15–30 min. Infants in both groups who failed the closure and had a persistent hsPDA after the first course of treatment received a second course of i.v. ibuprofen (10-5-5 mg/kg/day). Further pharmacological treatments and the need for surgical closure were decided on the basis of local protocol.

The allocation sequences consist of computer-generated random numbers. Since the frequency of the PDA is inversely related to the gestational age, the inclusion of patients was balanced in each treatment group according to the following gestational ages: 25^+0^–27^+6^ weeks or 28^+0^–31^+6^ weeks.

Daily clinical care of enrolled patients was performed by attending physicians in accordance with the common practice at each center. Daily fluid intake was started with 70–80 mL/kg and gradually increased by 10–20 mL/kg/day on the basis of changes in body weight, serum sodium concentrations, and osmolality, with a target intake of 150–160 mL/kg at the end of the first week of life. In case of systemic hypotension refractory to fluid replacement therapy, dopamine and/or dobutamine treatment were provided. For the treatment of RDS, infants received oxygen therapy, respiratory support, and rescue surfactant treatment in order to achieve the following targets: PaO_2_ 50–60 mmHg, PaCO_2_ < 65 mmHg, pH > 7.20, and SpO_2_ 90–95%.

Echocardiography was repeated every 24 h during the first treatment course, 24 h after the last dose of the treatment, at follow-up visits, and in case of clinical suspected PDA re-opening. Cardiac ultrasound was performed by expert personnel, specifically a pediatric cardiologist or a neonatologist who has achieved adequate expertise in newborn heart ultrasound, who were blinded to the study and treatment groups. More persons performed cardiac ultrasounds in each participating centers. Further details about respiratory management and timing for routine blood analysis were previously reported [23].

### Outcomes

The primary outcome of the study was the closure of hsPDA after the first course of treatment with paracetamol in comparison with ibuprofen.

Secondary outcomes were the constriction of hsPDA after the first course of treatment with paracetamol in comparison with ibuprofen (constricted DA was defined as closed DA or not hsPDA), the closure of hsPDA and the constriction rate after the second course of treatment with ibuprofen, and the re-opening rate and the incidence of need for surgical closure 30 days after the enrollment.

### Treatment emergent adverse effects

Laboratory tests were performed at patients’ screening, at the end of first and second course of treatment, and during the follow-up visits at 7 and 30 (± 2) days after the enrollment. Clinical laboratory tests included a count of red blood cells, white blood cells, and platelets, serum value measurement of hemoglobin/hematocrit, creatinine, urea nitrogen, total bilirubin, total proteins, liver enzymes, sodium, potassium, and calcium. For study purposes, renal failure was defined as serum creatinine concentration > 1.5 mg/dL and urine output < 1 mL/kg/h during a 24-h collection period. Liver failure was defined as elevated liver enzymes more than two times the upper boundary of the normal range (ALT 6–50 U/L; AST 35–140 U/L) [21]. Necrotizing enterocolitis (NEC) and isolated gastrointestinal perforation occurring within 30 days after the enrollment were also recorded.

### Further collected data

The following data were recorded for each infant: gestational age, birth weight, gender, mode of delivery, Apgar score at 5 min, main maternal pathologies, antenatal steroid treatment, and vital signs, such as heart rate, systolic, diastolic, and mean arterial blood pressure at the start of treatment, peak FiO_2_ and mean airway pressure values, need for non-invasive (nasal continuous airway pressure (NCPAP), biphasic positive airway pressure (BiPAP), nasal intermittent mandatory ventilation (N-IMV), humidified high flow nasal cannula (HHFNC)) and invasive respiratory support (patient triggered ventilation (PTV), including synchronized intermittent positive pressure ventilation (SIPPV), synchronized intermittent mandatory ventilation (SIMV), pressure support ventilation (PSV) or high frequency oscillatory ventilation (HFOV)), need for surfactant treatment, and adverse events. We also reported the occurrence of sepsis, IVH, periventricular leukomalacia (PVL), BPD, retinopathy of prematurity (ROP), NEC, length of hospital stay, and mortality.

Diagnosis of sepsis was based on clinical and laboratory data (white cell count, C-reactive protein concentration) and confirmed by positive blood cultures [24]. IVH was graded according to a Papile classification [25]. The diagnosis of PVL was performed in the presence of cystic areas detected by cerebral ultrasonography at 40-week post-conception birth [26]. BPD was defined as oxygen requirement at 36 weeks of post-menstrual age [27]. ROP was graded according to the international classification of retinopathy of prematurity [28]. NEC was diagnosed in agreement with classical Bell’s criteria [29].

All study data were collected on a web-based electronic case report form, specifically designed for this study.

### Statistical analysis

Assuming a 25% ibuprofen failure rate in closing hsPDA [30] and a 5% paracetamol failure rate [23] (improvement of 20%) after a 3-day course of treatment, we calculated that a sample size of 49 patients per group was necessary to determine a statistically significant decrease of 20% in the failure rate in the paracetamol group, at an alpha level of 5% two sided and with a power of 80%. Hypothesizing a 10% dropout rate of patients who did not complete the first course of treatment, we planned to enroll 55 infants in each group.

The following populations were defined for statistical analysis: the modified intention-to-treat (m-ITT) population as all randomized patients completing the first treatment course, having baseline and day 3 echocardiographic assessment, the per protocol (PP) population as patients from the m-ITT population with no major protocol violations, and the safety population (SP) as all patients who took at least one dose of study medication.

Analysis of the primary and secondary end points of the study was carried out on the m-ITT and PP populations, while safety and tolerability assessments were carried out on the SP population.

Clinical characteristics of infants in the paracetamol and ibuprofen groups were described using mean value and standard deviation, median value and interquartile range (IQR), or frequencies and percentage. Normality of data distribution was assessed by Shapiro–Wilk’s test. Parametric continuous variables were analyzed by the Student’s “*t*” test or by Wilcoxon rank sum test in case of deviation from normality assumptions. Categorical variables were compared using the *χ*^2^ test or Fisher’s exact test. *P* < 0.05 was considered statistically significant.

A multiple logistic regression analysis was performed using backward variable selection to assess the potential independent effect of the first course of paracetamol vs. ibuprofen, 25–27 vs. 28–31 weeks of gestational age, and peak FiO_2_ > 0.25 vs. ≤ 0.25 on the closure of PDA. These variables were selected because there were no differences between study groups at univariate analysis, but gestational age and the peak of FiO_2_ have been previously found to be correlated with the risk of developing hsPDA [19]. The goodness of fit was evaluated using the Hosmer–Lemeshow test. Effect estimates were expressed as odds ratio (OR) with profile likelihood-based 95% confidence limits.

## Results

**Fig. 1:**
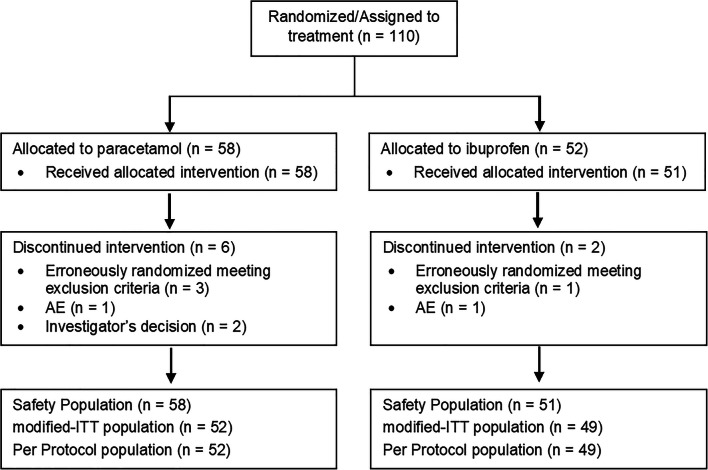
Patient disposition and statistical study populations: m-ITT population, PP population, and SP

The study was carried out from December 2015 to January 2019. Figure 1 shows the patient disposition with the number of m-ITT, PP, and SP populations. Since no major protocol violations were detected, the m-ITT and PP populations matched. Primary and secondary end points were evaluated in 52 and 49 infants (m-ITT population) who received paracetamol or ibuprofen as first treatment course, respectively. Both groups had comparable infant clinical and maternal characteristics (Table 1). TEAEs were evaluated in the SP population (58 and 51 infants who received at least one dose of paracetamol or ibuprofen, respectively).

**Table 1:** Clinical characteristics of infants in the paracetamol and ibuprofen groups

Variable	Paracetamol (*n* = 52)	Ibuprofen (*n* = 49)	*P*
Gestational age (weeks)	28.2 + 1.4	28.4 + 2.0	0.495
25–27 weeks	18 (35)	17 (35)	0.993
28–31 weeks	34 (65)	32 (65)	0.993
Birth weight (g)	1022 + 266	1068 + 278	0.423
< 10° percentile	8 (15)	11 (22)	0.448
Male	21 (40)	21 (43)	0.801
Apgar score at 5 min	8 (8–8)	8 (8–8)	0.845
Antenatal steroids	41 (79)	40 (82)	0.805
Singleton	24 (46)	31 (63)	0.084
Cesarean section	45 (87)	37 (76)	0.156
Abruptio placentae	4 (8)	1 (2)	0.363
Hypertension disorders of pregnancy	13 (25)	9 (18)	0.476
pPROM	5 (10)	5 (10)	1.000
Gestational diabetes	4 (8)	7 (14)	0.349
Peak FiO_2_	0.29 + 0.11	0.27 + 0.07	0.852
Peak MAP (cmH_2_O)	8.9 + 3.3	8.8 + 3.2	0.945
RDS	49 (94)	43 (88)	0.254
Surfactant	30 (58)	31 (63)	0.684
Non-invasive respiratory support	43 (83)	39 (80)	0.690
Mechanical ventilation	8 (15)	6 (12)	0.866
IVH	3 (6)	4 (8)	0.636
≥ 3 grade IVH	0	1 (2)	0.301
BPD^a^	3 (16)	4 (12)	0.709
PVL	0	0	N/A
NEC	2 (4)	1 (2)	0.593
Gastrointestinal perforation	1 (2)	0	0.329
ROP^b^	1 (5)	3 (8)	0.610
≥ 3 grade ROP	0	0	N/A
Early-onset sepsis	3 (6)	0	0.088
Late-onset sepsis	9 (17)	14 (29)	0.177
Hospital stay duration (d)	57.6 + 13.2	59.9 + 15.5	0.633
Mortality	3 (6)	1 (2)	0.337

Data presented as mean ± SD, rate (%), or median (IQR)

*pPROM* preterm premature rupture of membrane, *MAP* mean airway pressure, *RDS* respiratory distress syndrome, *IVH* intraventricular hemorrhage, *BPD* bronchopulmonary dysplasia, *PVL* periventricular leukomalacia, *NEC* necrotizing enterocolitis, *ROP* retinopathy of prematurity

^a^Calculated from available data of 19 patients in paracetamol and 32 in ibuprofen

^b^Calculated from available data of 21 patients in paracetamol and 36 in ibuprofen

Heart rate, systolic, diastolic and mean arterial blood pressure, hemoglobin, and platelet count at the start of treatment were similar in the paracetamol and ibuprofen group. Treatment of hsPDA was started at 46 ± 15 and 46 ± 16 h (*P* = 0.877) of life in the paracetamol and ibuprofen group, respectively (Table 2).

**Table 2:** Clinical characteristics of infants in the paracetamol and ibuprofen groups at enrolment. Mean + SD, rate and (%), or median and (IQR)

	Paracetamol (*n* = 52)	Ibuprofen (*n* = 49)	*P*
Age at enrolment (h)	46 ± 15	46 ± 16	0.877
Heart rate (bpm)	157 ± 11	156 ± 11	0.693
Systolic arterial blood pressure (mmHg)	56 ± 11	56 ± 10	0.681
Diastolic arterial blood pressure (mmHg)	33 ± 9	31 ± 7	0.300
Mean arterial blood pressure (mmHg)	42 ± 9	40 ± 8	0.205
Hemoglobin (g/dL)	14.8 ± 2.7	15.8 ± 2.6	0.064
Platelets (10^9^/L)	200.0 ± 108.3	205.1 ± 87.2	0.790

Paracetamol was less effective in closing hsPDA than ibuprofen (52 vs. 78%; *P* = 0.026), but the success rate in constricting the DA was similar (81 vs. 90%; *P* = 0.202). The effectiveness of the second course of treatment with ibuprofen was also similar in infants who had been previously treated with paracetamol or ibuprofen, either in closing (40 vs. 50%; *P* = 0.452) and in constricting hsPDA (70 vs. 100%; *P* = 0.216) (Table 3).

**Table 3:** Primary and secondary outcomes of the study

	**Paracetamol (*n*** **= 52)**	**Ibuprofen (*n*** **= 49)**	***P***
First course of treatment			
Closed DA	27 (52)	38 (78)	0.026
Not hsPDA	15 (29)	6 (12)	0.039
Constricted DA (closed or not hsPDA)	42 (81)	44 (90)	0.202
hsPDA	10 (19)	5 (10) ^a^	0.202
	***n*** **= 10**	***n*** **= 5**	
Second course of treatment with ibuprofen^b^			
Closed DA	4 (40)	2 (50)	0.452
Not hsPDA	3 (30)	2 (50)	0.696
Constricted DA (closed or not hsPDA)	7 (70)	4 (100)	0.216
hsPDA	3 (30)	0 (0)	0.088
Re-opening within 30 days of life^c^	14 (36)	8 (19)	0.078
Surgical closure within 30 days of life^c^	0	1 (2)	0.338

Data presented as rate (%)

*DA* ductus arteriosus, *hsPDA* hemodynamically patent ductus arteriosus

^a^One patient in ibuprofen group was not treated with the second course of treatment as per physician’s decision

^b^In both groups

^c^In infants who had constricted DA (closed + not hsPDA) after the first course of treatment: calculated from available data of 39 patients in paracetamol group and 43 in ibuprofen group

The re-opening rate of the DA was similar (36 vs. 19%; *P* = 0.078) in infants both in the paracetamol and ibuprofen groups, as the need for surgical closure (0 vs. 2%; *P* = 0.338) (Supplemental Table S1 (online)).

**Fig. 2:**
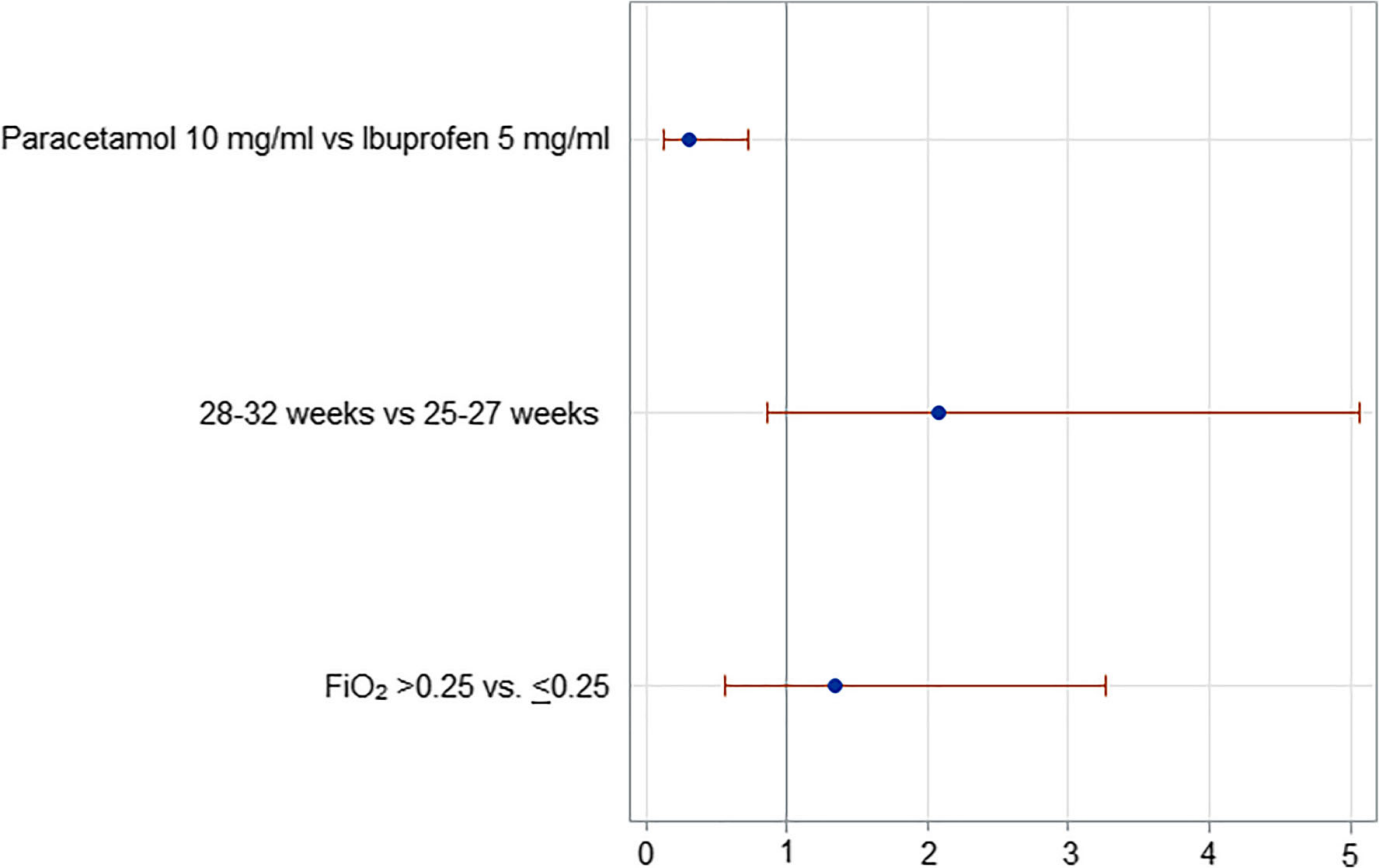
Logistic regression analysis: Closed DA vs. not hsPDA + hsPDA

**Fig. 3:**
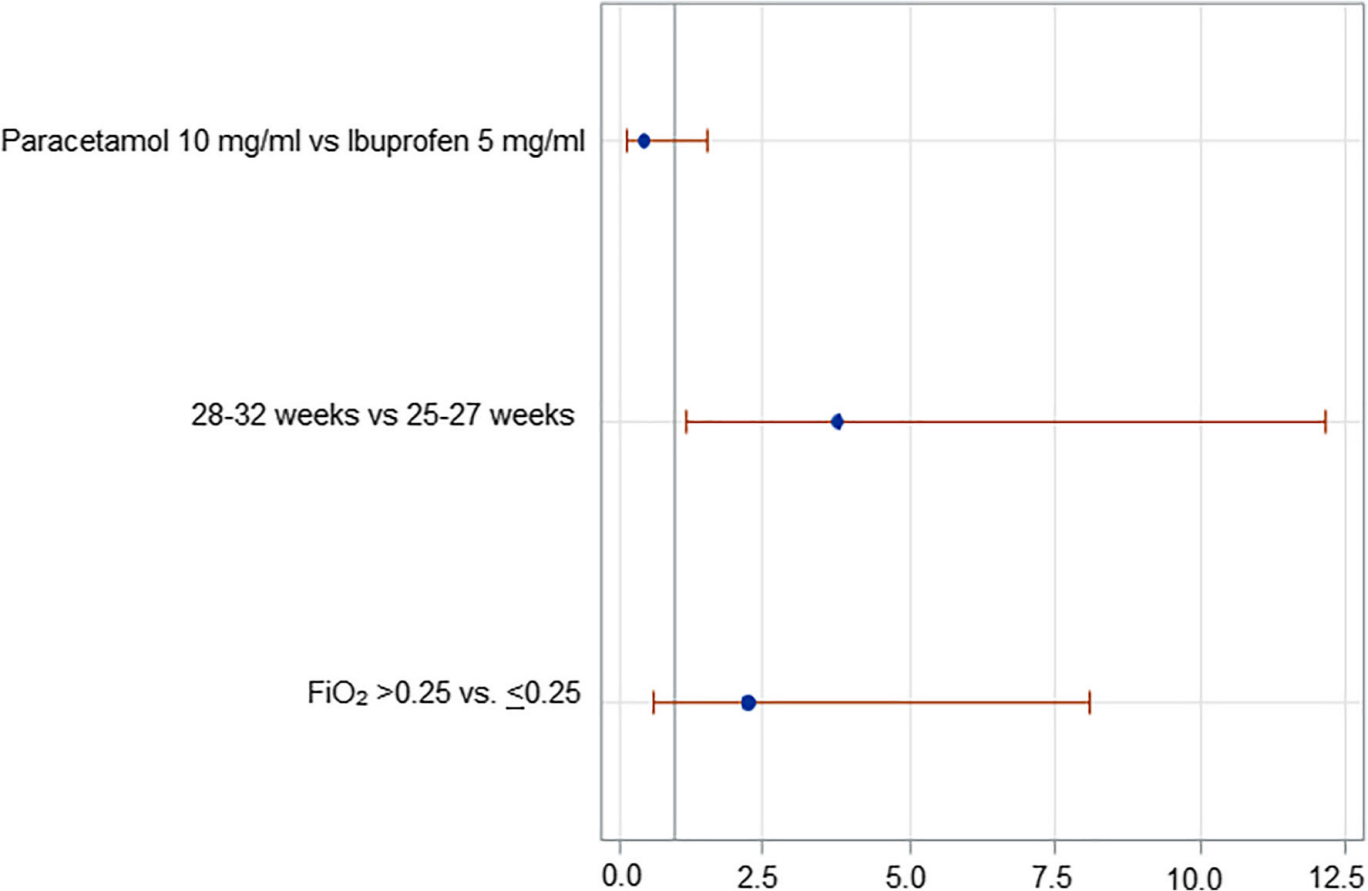
Logistic regression analysis: Closed DA + not hsPDA vs. hsPDA

Logistic regression analysis demonstrated that the use of paracetamol decreased the likelihood of PDA closure after the first course of treatment in comparison with ibuprofen (OR 0.30; Cl 95% 0.13–0.73) but did not affect the likelihood of PDA constriction (OR 0.46; Cl 95% 0.14–1.51). Patients’ gestational age and peak FiO_2_ did not affect PDA closure rate (Fig. 2), while 25–27 weeks of gestational age decreased the likelihood of PDA constriction in comparison with 28–32 weeks of gestational age (Fig. 3).

Occurrence of adverse events was similar in paracetamol and ibuprofen groups and is detailed in Supplemental Table S1 (online). Occurrence of renal and liver failure, NEC, and gastrointestinal perforation was very low and did not differ between the groups (Supplemental Table S2 (online)). None of the patients had to discontinue the treatment because of liver or renal toxicity. Bleeding disorders and white cells, red cells, and platelet counts did not differ between the groups (Supplemental Table S3 (online)), nor did the occurrence of prematurity complications, hospital stay, or mortality (Supplemental Table S1 (online)).

Four infants died for NEC (*n* = 2, in the paracetamol group) or intestinal volvulus (*n* = 2, one in both the groups).

## Discussion

In this study, we assessed the efficacy of i.v. paracetamol in comparison with i.v. ibuprofen for the treatment of hsPDA in preterm infants and we found that paracetamol was less effective in closing hsPDA than ibuprofen. Our result disagrees with previous findings by Dang [12] and Oncel [13], who found that oral paracetamol was effective as oral ibuprofen in closing hsPDA. These different results might be due to the different route of administration, even if paracetamol serum levels after 48 h of an i.v. course have been reported to be higher than those achieved after 48 h of an oral course (this could suggest the lack of correlation between paracetamol serum level and its closure activity) [31]. On the other hand, also oral ibuprofen has been found more effective in closing PDA than i.v. ibuprofen due to unknown mechanisms [32]. It is more difficult to explain the discordance between our findings and those of El-Mashad et al. [14], who found that i.v. paracetamol was effective as i.v. ibuprofen and indomethacin in closing hsPDA in a large RCT (*n* = 300): both studies used the same course of paracetamol and ibuprofen, at the same post-natal age, with the only relevant difference being that they enrolled more immature infants than our study. However, other studies are in agreement with our results: Roofthooft and Alan reported case series in which i.v. paracetamol failed to close hsPDA [33, 34], and a recent large retrospective study (*n* = 842) reported that the use of paracetamol as first-treatment course increased the risk of hsPDA closure failure in comparison with ibuprofen both in infants born at 23–24 and 25–28 weeks of gestation [18]. Moreover, these data are consistent with the results of a recent study in isolated mouse DA demonstrating that paracetamol has a lower effect in constricting the DA and decreasing the prostaglandin synthesis than indomethacin [35].

It is relevant that the success rate in constricting hsPDA (defined as closed DA or not hsPDA) was similar in infants treated with paracetamol or ibuprofen (81 vs. 90%). This result is in discordance with the study by Liebowitz et al. who found that paracetamol has a lower hsPDA constriction effect than ibuprofen and indomethacin [18]. However, this study was a retrospective secondary data analysis of the multicenter PDA-TOLERATE trial in which infants were treated later than in our trial (during the second week of life) and with both oral and i.v. paracetamol. In any case, we believe that our findings are very important because they show that, although paracetamol was less effective than ibuprofen in closing hsPDA, the same percentage of infants were exposed to the second course of treatment in both groups. Moreover, we demonstrated that starting treatment of hsPDA with paracetamol did not negatively affect the effectiveness of the second course of treatment with ibuprofen, both in closing and constricting hsPDA, in the occurrence of PDA re-opening, and in the need for surgical closure. Thus, in light of the well-known better safety profile of paracetamol in comparison with ibuprofen and indomethacin [15, 16], our results can support the use of paracetamol as a first choice in the treatment of hsPDA and, in the future, the re-evaluation of a prophylactic approach of the hsPDA, which is not largely diffused due to the adverse effects of ibuprofen and indomethacin.

We observed that a second pharmacological course with ibuprofen was effective in closing hsPDA refractory to the first treatment course in the preterm infant. This result is in agreement with previous studies which demonstrated that repeated courses of ibuprofen are an effective and safe alternative for surgical closure and should be considered after failure of the first course of ibuprofen [36, 37].

We found that infants born at 25–27 weeks of gestational age had lower likelihood of PDA constriction than infants born at 28–32 weeks of gestational age. These results confirm previous findings that hsPDA occurrence is inversely related to gestational age probably due to the increase of DA reactivity to oxygen and decrease of circulating vasodilator concentration occurring as gestational age progresses [19]. Moreover, it has been reported that also reactive oxygen species (ROS) and isoprostanes (IsoPs) have a role in DA closure [38]. In particular, IsoPs can have both constrictive and dilatory effects on the DA mediated by the activation of thromboxane A2 (TxA2) receptor or prostaglandin E2 receptor 4 (EP4), respectively [38]. With increasing maturity, the balance between EP4 and TxA2 receptors shifts in favor of the contractile effects of TxA2 stimulation, and this contributes to explain the higher rate of hsPDA and its refractoriness to pharmacological closure in more immature infants.

In our study, we also collected data regarding adverse effects of drugs, the occurrence of which was similar for paracetamol and ibuprofen. Both were substantially safe, but the size of our population did not allow firm conclusions on this issue.

A limitation of the study was the lack of a double-blind design due to the different number of daily doses of paracetamol and ibuprofen. However, the primary end point (closure of hsPDA) was evaluated through objective echocardiographic cardiovascular measurements and we are confident that this could contribute to limit the risk of bias. Another limitation is that our echocardiographic criteria for hsPDA diagnosis (i.e., left atrium-to-aortic root ratio > 1.3 or a ductal size > 1.5 mm), although they are widely diffused, may have significant variability between observers. Moreover, we could not evaluate the effect of hsPDA closure or constriction on patients’ main outcomes, such as mortality and BPD, due to the lack of a control group using a non-interventional conservative management of PDA. However, these outcomes were not the objectives of our study. We recognize that our study has a relative lack of novelty compared with other studies; however, we believe it may be very useful for future meta-analyses. We did not collect serial data on FiO_2_ and pO_2_ during treatment and, therefore, we could not evaluate the effect of oxygen therapy on PDA closure.

## Conclusions

We found that the first course of treatment of hsPDA with i.v. paracetamol was less effective than i.v. ibuprofen in closing hsPDA in preterm infants with gestational age ≥ 25 weeks but has a similar constriction effect and its use was associated with the same hsPDA outcome, i.e., the same need for a second course of treatment, re-opening rate, and surgical closure requirement. Both drugs had similar safety profile. These results can support the use of i.v. paracetamol as a first-choice drug for the treatment of hsPDA.

## Electronic supplementary material

ESM 1 (DOCX 28 kb)

ESM 2 (DOCX 29 kb)

ESM 3 (DOCX 46 kb)

ESM 4 (DOC 352 kb)

ESM 5 (DOC 49 kb)

## Abbreviations

ALT: Alanine aminotransferase
AST: Aspartate aminotransferase
BPD: Bronchopulmonary dysplasia
DA: Ductus arteriosus
hsPDA: Hemodynamically significant patent ductus arteriosus
IQR: Interquartile range
i.v.: Intravenous
IVH: Intraventricular hemorrhage
m-ITT: Modified intention-to-treat
NEC: Necrotizing enterocolitis
NSAIDs: Non-steroidal anti-inflammatory drugs
OR: Odds ratio
PP: Per protocol
PVL: Periventricular leukomalacia
RCT: Randomized controlled trial
RDS: Respiratory distress syndrome
ROP: Retinopathy of prematurity
SP: Safety population
TEAEs: Treatment emergent adverse events
