# Supplementary material for: Intravenous paracetamol in comparison with ibuprofen for the treatment of patent ductus arteriosus in preterm infants: a randomized controlled trial
Source: Eur J Pediatr. 2020 Sep 4;180(3):807–16. doi: 10.1007/s00431-020-03780-8 (PMC7886841; doi:10.1007/s00431-020-03780-8)
Supplement: Supplementary file 1 — (DOCX 28 kb) [file 431_2020_3780_MOESM1_ESM.docx]

**Supplemental Table S1 (online).** Overall summary of TEAEs in Safety Population.

| **Variable** | **Paracetamol**  **(n = 58)** | **Ibuprofen**  **(n = 51)** | **P** |
| --- | --- | --- | --- |
| **No. of Adverse Events** | 171 (46.6) | 196 (53.4) | 0.065 |
| **Number of subjects with at least one AE** | 44 (75.9) | 43 (84.3) | 0.273 |
| **Number of subjects with ADRs** | 2 (3.4) | 6 (11.8) | 0.097 |
| **Number of subjects with at least one severe AE** | 7 (12.1) | 5 (9.8) | 0.706 |

Data presented as rate (%).

*ADR*, adverse drug reaction; *AE*, adverse event.
