# Supplementary material for: Intravenous paracetamol in comparison with ibuprofen for the treatment of patent ductus arteriosus in preterm infants: a randomized controlled trial
Source: Eur J Pediatr. 2020 Sep 4;180(3):807–16. doi: 10.1007/s00431-020-03780-8 (PMC7886841; doi:10.1007/s00431-020-03780-8)
Supplement: Supplementary file 2 — (DOCX 29 kb) [file 431_2020_3780_MOESM2_ESM.docx]

**Supplemental Table S2 (online).** Incidence of renal failure, liver failure and gastrointestinal complications within 30 days after enrollment.

| **Variable** | **Paracetamol**  **(n = 52)** | **Ibuprofen**  **(n = 49)** | **P** |
| --- | --- | --- | --- |
| **Renal failure^a^** | 0 | 0 | N/A |
| **Liver failure^b^** | 1 (2) | 0 | 0.615 |
| **NEC** | 1 (2) | 1 (2) | 0.966 |
| **Gastrointestinal perforations** | 1 (2) | 0 | 0.329 |

Data presented as rate (%).

*NEC*, necrotizing enterocolitis.

^a^Renal failure: serum creatinine concentration > 1.5 mg/dl (132 μmol/l) and urine output <1 ml/kg/h during a 24-h collection period.

^b^Liver failure: ALT and AST >2 times the upper boundary of the normal range (ALT: 6-50 U/L; AST: 35-140 U/L).
