# Supplementary material for: Intravenous paracetamol in comparison with ibuprofen for the treatment of patent ductus arteriosus in preterm infants: a randomized controlled trial
Source: Eur J Pediatr. 2020 Sep 4;180(3):807–16. doi: 10.1007/s00431-020-03780-8 (PMC7886841; doi:10.1007/s00431-020-03780-8)
Supplement: Supplementary file 3 — (DOCX 46 kb) [file 431_2020_3780_MOESM3_ESM.docx]

**Supplemental Table S3 (online).** Distribution of TEAEs at 30 days in Safety Population.

| **System Organ Class** | **Total** | **Paracetamol**  **(n = 146)** | **Ibuprofen**  **(n = 144)** |
| --- | --- | --- | --- |
| **Blood and lymphatic system disorders** | Anaemia | 7 (4.8) | 5 (3.5) |
|  | Anaemia neonatal | 11 (7.5) | 9 (6.3) |
|  | Coagulation disorder neonatal | 1 (0.7) |  |
|  | Coagulopathy | 1 (0.7) | 1 (0.7) |
|  | Hypercoagulation |  | 1 (0.7) |
|  | Leucocytosis | 1 (0.7) | 1 (0.7) |
|  | Leukopenia | 1 (0.7) | 1 (0.7) |
|  | Thrombocytopenia | 2 (1.4) | 2 (1.4) |
|  | Thrombocytosis |  | 3 (2.1) |
| **Cardiac disorders** | Atrial thrombosis | 1 (0.7) | 1 (0.7) |
|  | Bradycardia neonatal |  | 2 (1.4) |
|  | Cardiac failure | 1 (0.7) |  |
|  | Left ventricular hypertrophy |  | 1 (0.7) |
|  | Neonatal tachycardia |  | 1 (0.7) |
|  | Ventricular hypertrophy |  | 1 (0.7) |
| **Congenital, familial and genetic disorders** | Congenital hypothyroidism | 1 (0.7) | 2 (1.4) |
|  | Ventricular septal defect |  | 1 (0.7) |
| **Gastrointestinal disorders** | Abdominal distension | 2 (1.4) | 1 (0.7) |
|  | Constipation |  | 1 (0.7) |
|  | Enteritis | 1 (0.7) |  |
|  | Gastric haemorrhage | 2 (1.4) | 3 (2.1) |
|  | Gastritis |  | 1 (0.7) |
|  | Gastroesophageal reflux disease |  | 1 (0.7) |
|  | Haematochezia |  | 1 (0.7) |
|  | Intestinal perforation | 1 (0.7) |  |
|  | Necrotising colitis | 1 (0.7) |  |
|  | Reflux gastritis | 2 (1.4) |  |
|  | Volvulus | 1 (0.7) |  |
|  | Vomiting |  | 4 (2.8) |
| **General disorders and administration site conditions** | Fever neonatal | 1 (0.7) |  |
|  | Oedema | 1 (0.7) | 1 (0.7) |
|  | Puncture site haemorrhage | 1 (0.7) |  |
| **Hepatobiliary disorders** | Hypertransaminasaemia | 1 (0.7) |  |
|  | Jaundice | 3 (2.1) | 1 (0.7) |
|  | Neonatal cholestasis | 1 (0.7) |  |
| **Infections and infestations** | Conjunctivitis | 3 (2.1) | 6 (4.2) |
|  | Ear infection | 1 (0.7) |  |
|  | Eye infection bacterial | 1 (0.7) |  |
|  | Ophthalmia neonatorum | 2 (1.4) | 1 (0.7) |
|  | Pneumonia |  | 1 (0.7) |
|  | Rotavirus infection | 1 (0.7) |  |
|  | Sepsis | 9 (6.2) | 7 (4.9) |
|  | Sepsis neonatal | 2 (1.4) | 4 (2.8) |
|  | Staphylococcal sepsis | 1 (0.7) |  |
| **Injury, poisoning and procedural complications** | Medication error | 3 (2.1) |  |
|  | Skin abrasion |  | 1 (0.7) |
| **Investigations** | Cardiac murmur |  | 1 (0.7) |
| **Metabolism and nutrition disorders** | Acidosis | 3 (2.1) | 1 (0.7) |
|  | Dehydration | 1 (0.7) |  |
|  | Feeding disorder of infancy or early childhood | 8 (5.5) | 4 (2.8) |
|  | Hypercalcaemia | 1 (0.7) | 1 (0.7) |
|  | Hyperglycaemia | 3 (2.1) | 1 (0.7) |
|  | Hypernatraemia |  | 1 (0.7) |
|  | Hypocalcaemia | 1 (0.7) |  |
|  | Hypoglycaemia | 3 (2.1) |  |
|  | Hypoglycaemia neonatal |  | 1 (0.7) |
|  | Hypokalaemia | 8 (5.5) | 6 (4.2) |
|  | Hyponatraemia | 14 (9.6) | 18 (12.5) |
|  | Metabolic acidosis | 19 (13.0) | 18 (12.5) |
|  | Metabolic disorder |  | 1 (0.7) |
|  | Neonatal hyponatraemia |  | 3 (2.1) |
| **Psychiatric disorders** | Agitation | 2 (1.4) | 1 (0.7) |
| **Renal and urinary disorders** | Acute kidney injury | 1 (0.7) |  |
|  | Oliguria |  | 3 (2.1) |
|  | Renal disorder |  | 1 (0.7) |
| **Respiratory, thoracic and mediastinal disorders** | Apnoea neonatal | 1 (0.7) | 3 (2.1) |
|  | Bronchopulmonary dysplasia |  | 1 (0.7) |
|  | Lung infiltration |  | 1 (0.7) |
|  | Neonatal respiratory acidosis | 2 (1.4) |  |
|  | Neonatal respiratory alkalosis | 1 (0.7) | 1 (0.7) |
|  | Neonatal respiratory failure | 2 (1.4) | 1 (0.7) |
|  | Pulmonary haemorrhage | 1 (0.7) |  |
|  | Pulmonary oedema neonatal | 1 (0.7) |  |
|  | Respiratory acidosis |  | 3 (2.1) |
|  | Respiratory failure | 1 (0.7) |  |
|  | Stridor | 1 (0.7) |  |
|  | Tachypnoea | 1 (0.7) | 1 (0.7) |
| **Skin and subcutaneous tissue disorders** | Skin discolouration |  | 1 (0.7) |
|  | Skin lesion | 1 (0.7) | 1 (0.7) |
| **Surgical and medical procedures** | Astringent therapy |  | 1 (0.7) |
| **Vascular disorders** | Haemorrhage |  | 1 (0.7) |
|  | Hyperaemia |  | 1 (0.7) |
|  | Hypotension | 1 (0.7) |  |
|  | Neonatal hypotension | 1 (0.7) |  |
|  | Peripheral ischaemia |  | 1 (0.7) |

Data presented as rate (%).
