# Supplementary material for: Intravenous paracetamol in comparison with ibuprofen for the treatment of patent ductus arteriosus in preterm infants: a randomized controlled trial
Source: Eur J Pediatr. 2020 Sep 4;180(3):807–16. doi: 10.1007/s00431-020-03780-8 (PMC7886841; doi:10.1007/s00431-020-03780-8)
Supplement: Supplementary file 5 — (DOC 49 kb) [file 431_2020_3780_MOESM5_ESM.doc]

**CONSORT 2010 Flow Diagram**

**Allocation**

**Analysis**

**Follow-Up**

**Enrollment**

Assessed for eligibility (n=110)

Excluded (n=0)

Safety Population (n = 58)

♦ Excluded from analysis (n=6): discontinuation of intervention

modified-ITT population (n = 52)

Per Protocol population (n = 52)

Discontinued intervention (n = 6)

- Erroneously randomized meeting exclusion criteria (n = 3)
- AE (n = 1)
- Investigator’s decision (n = 2)

Allocated to paracetamol (n=58)

♦ Received allocated intervention (n=58)

♦ Did not receive allocated intervention (give reasons) (n=0)

Discontinued intervention (n = 2)

- Erroneously randomized meeting exclusion criteria (n = 1)
- AE (n = 1)

Allocated to ibuprofen (n=52)

♦ Received allocated intervention (n=51)

♦ Did not receive allocated intervention (give reasons) (n=1) (erroneously randomized)

Safety Population (n = 51)

♦ Excluded from analysis (n=2): discontinuation of intervention

modified-ITT population (n = 49)

Per Protocol population (n = 49)

Randomized (n=110)
